# Development and Validation of a Prediction Rule for Growth Hormone Deficiency Without Need for Pharmacological Stimulation Tests in Children With Risk Factors

**DOI:** 10.3389/fendo.2020.624684

**Published:** 2021-02-03

**Authors:** Florencia Clément, Romina P. Grinspon, Daniel Yankelevich, Sabrina Martín Benítez, María Carolina De La Ossa Salgado, María Gabriela Ropelato, María Gabriela Ballerini, Ana C. Keselman, Débora Braslavsky, Patricia Pennisi, Ignacio Bergadá, Gabriela P. Finkielstain, Rodolfo A. Rey

**Affiliations:** ^1^ Centro de Investigaciones Endocrinológicas “Dr César Bergadá” (CEDIE), CONICET-FEI-División de Endocrinología, Hospital de Niños Ricardo Gutiérrez, Buenos Aires, Argentina; ^2^ Practia S.A., Buenos Aires, Argentina; ^3^ Fundación para el Desarrollo Argentino (FUNDAR), Buenos Aires, Argentina; ^4^ Hospital Dr Humberto J. Notti, Mendoza, Argentina; ^5^ Takeda Pharma, Buenos Aires, Argentina

**Keywords:** multiple pituitary hormone deficiencies, pituitary dysgenesis, short stature, growth failure, midline abnormalities

## Abstract

**Introduction:**

Practice guidelines cannot recommend establishing a diagnosis of growth hormone deficiency (GHD) without performing growth hormone stimulation tests (GHST) in children with risk factors, due to the lack of sufficient evidence.

**Objective:**

Our goal was to generate an evidence-based prediction rule to diagnose GHD in children with growth failure and clinically identifiable risk factors.

**Methods:**

We studied a cohort of children with growth failure to build the prediction model, and a second, independent cohort to validate the prediction rule. To this end, we assessed the existence of: pituitary dysgenesis, midline abnormalities, (supra)sellar tumor/surgery, CNS infection, traumatic brain injury, cranial radiotherapy, chemotherapy, genetic GHD, pituitary hormone deficiencies, and neonatal hypoglycemia, cholestasis, or hypogenitalism. Selection of variables for model building was performed using artificial intelligence protocols. Specificity of the prediction rule was the main outcome measure in the validation set.

**Results:**

In the first cohort (n=770), the resulting prediction rule stated that a patient would have GHD if (s)he had: pituitary dysgenesis, or two or more anterior pituitary deficiencies, or one anterior pituitary deficiency plus: neonatal hypoglycemia or hypogenitalism, or diabetes insipidus, or midline abnormalities, or (supra)sellar tumor/surgery, or cranial radiotherapy ≥18 Gy. In the validation cohort (n=161), the specificity of the prediction rule was 99.2% (95% CI: 95.6–100%).

**Conclusions:**

This clinical rule predicts the existence of GHD with high specificity in children with growth disorders and clinically identifiable risk factors, thus providing compelling evidence to recommend that GHD can be safely diagnosed without recurring to GHST in neonates and children with growth failure and specific comorbidities.

## Introduction

Growth is a good indicator of a child’s health, and growth failure prompts the pediatrician to search for nutrition disorders, subclinical chronic diseases, or hormone deficiencies. Growth hormone deficiency (GHD) is characterized by the insufficient production of growth hormone (GH), which leads to deficient insulin-like growth factor 1 (IGF1) synthesis and secretion leading to growth failure in children. An accurate diagnosis is crucial for timely initiation of treatment in order to optimize child growth and adult height and to avoid co-morbidities resulting in impaired quality of life (1).

Auxologic evaluation lies at the basis of the diagnosis of GHD in children (2–4), and the Growth Hormone Research Society clearly defined in its consensus guidelines released in year 2000 the clinical criteria that should prompt immediate investigation of GHD in childhood and adolescence (5). Many national endocrine societies have set up procedures to diagnose GHD, and the health authorities of several countries have established national or regional boards that review and monitor GH prescriptions (2, 6). Multiple GH stimulation tests (GHSTs) have been designed to evaluate GH sufficiency in children in an attempt to reach the most accurate diagnosis of GHD (7, 8). Although they have limitations, GHSTs are still used as the gold standard for the diagnosis of pediatric GHD in most countries (1, 8).

The guidelines of the US Pediatric Endocrine Society advocate for restricting GH testing (1). For instance, in newborns with hypoglycemia and/or neonatal cholestasis the diagnosis of GHD is an emergency, and GHSTs may be dangerous (9). In a child with growth failure, the presence of micropenis and cryptorchidism or of craniofacial midline abnormalities are other putative predictors of GH deficiency (1, 5). Acquired GHD may be suspected in patients with intracranial tumors, severe traumatic brain injury or cranial radiotherapy in whom a common co-morbidity is hypothalamic obesity, associated with blunted response during GHSTs (10). However, none of these studies provide predictive values that can guide medical decisions. Therefore, due to the lack of sufficient evidence, the guidelines cannot recommend establishing the diagnosis of GHD without GHSTs in patients with these conditions (1, 11).

Pharmacological GHSTs remain a standard practice in pediatric patients—usually due to requirements from health systems (6)—even in children with clearly identifiable potential risk factors for GHD (12–14) in whom the implementation of GHSTs might be considered redundant (1, 6, 15). The aim of the present study was to assess predictors of GHD in children with growth failure by analyzing a large cohort of pediatric patients in whom GHSTs had been performed in a tertiary referral center. Our primary objective was to develop and validate an accurate clinical prediction rule with high enough specificity to allow confirmation of the diagnosis of GHD in children without recurring to GHSTs. We, therefore, designed and validated a multivariable prediction model in accordance with the Transparent Reporting of a Multivariable Prediction Model for Individual Prognosis or Diagnosis (TRIPOD) statement (16).

## Patients and Methods

### Study Design and Data Sources

We performed a study designed to develop and validate a prediction rule to diagnose GHD with the highest specificity rate in children with growth failure and clinically identifiable risk factors, who underwent GHSTs. We analyzed clinical and biochemical characteristics and brain imaging findings in all patients younger than 18 years of age who underwent GHSTs at the Division of Endocrinology of the Hospital de Niños Ricardo Gutiérrez, a tertiary pediatric public hospital in the city of Buenos Aires, Argentina, between August 1, 2004 and July 31, 2014. Additionally, we validated the predictive rule in a second, independent cohort including GHSTs performed between February 1, 2017 and January 31, 2019.

We included GHSTs performed in patients who:

1. Met the criteria required for performing a GHST according to the *Summary Statement of the Growth Hormone Research Society* (5), as follows:
  - Severe short stature, defined as a height >3 SD below the mean, or
  - Height >1.5 SD below the mid-parental height, or
  - Height >2 SD below the mean and a height velocity over 1 year >1 SD below the mean for chronological age, or a decrease in height SD of >0.5 over 1 year in children over 2 years of age, or
  - In the absence of short stature, a height velocity >2 SD below the mean over 1 year or >1.5 SD sustained over 2 years, or
  - Signs indicative of an intracranial lesion, or
  - Signs of multiple pituitary hormone deficiency, or
  - Neonatal symptoms and signs of GHD.
2. Underwent two sequential GHSTs (arginine-clonidine), or only one test in patients weighing <10 kg.

We excluded GHSTs performed in patients:

1. With incomplete medical records.
2. With poorly controlled chronic disorders (e.g., hypothyroidism, or gastrointestinal, immunological, nephrogenic or hematological diseases).
3. In whom the GHST was performed for re-testing reasons.

### Case Ascertainment, Control Selection and Predictors

GHD (case) was diagnosed when maximal stimulated GH concentration was <6.1 ng/ml (from 2004 to 2011) (17) or <4.7 ng/ml (from 2012 to 2019) (18) during sequential arginine-clonidine tests, or a single arginine test in children weighing <10 Kg, according to previously validated cut-off values (19). Controls, i.e., non-GHD patients meeting the criteria required for GHST, were all the patients with at least one GH peak ≥6.1 ng/ml (2004–2011) or ≥4.7 ng/ml (since 2012).

To develop the model, we tested 15 potentially predictive dichotomous variables, as defined in **Table 1**, and auxologic data and IGF1 and IGFBP3 serum levels, as continuous variables.

**Table 1:** Definitions of predictors used in the model building.

Predictor	Operational definition	Reference
Pituitary dysgenesis	MRI of the hypothalamic-pituitary region, pre- and post-gadolinium enhanced T1- and T2 weighted images, with at least two of the following: anterior pituitary hypoplasia or aplasia, interrupted or hypoplastic stalk, ectopic or absent posterior lobe (including empty sella)	(15)
Clinical or radiological craniofacial midline abnormalities	Cleft lip and palate, single central incisor, agenesis of the nasal septum, septo-optic dysplasia, Rieger syndrome, holoprosencephaly, transsphenoidal myelomeningocele, hydrocephalus, Chiari type 1 malformation	
Sellar or suprasellar tumor/surgery	Imaging study of the CNS indicating the existence of a mass in the sellar or suprasellar region, or surgical report indicating compromise of sellar or suprasellar region, except for pituitary microadenoma	
Central nervous system infection	Meningitis, meningoencephalitis, encephalitis, pyogenic ventriculitis	
Severe traumatic brain injury	Glasgow Coma Scale score ≤8	(20)
Cranial radiotherapy	≥18 Gy	(21)
Chemotherapy	Use of mono- or poly-chemotherapy for at least 6 months	
Familial or sporadic GHD of genetic etiology	Index case and one or more first-degree relatives with GHD in the family, or index case with a pathogenic mutation	
TSH deficiency	Serum basal free T4 < 0.8 ng/dl with TSH ≤ 10 mIU/l in patients under 2 months of age and ≤ 6.5 mIU/l in older infants	(22)
ACTH deficiency	Serum basal cortisol < 6.5 µg/dl with low or normal plasma ACTH	(22)
Prolactin deficiency	Serum basal prolactin < 2.5^th^ centile for age and sex	(22)
Central diabetes insipidus	Polyuria associated with a urinary:plasma osmolarity ratio <1.5 and plasma osmolarity >300 mosm/l	(22)
Neonatal persistent hypoglycemia	Plasma glucose <50 mg/dl (= 2.8 mmol/l) days 3−28 of age (i.e., the period of transitional glucose regulation of postnatal days 1−2 has passed)	(23)
Neonatal cholestatic jaundice	Conjugated bilirubin/total bilirubin >0.15	(24)
Neonatal hypogenitalism	At least two of the following: micropenis defined as penile length <–2.5 standard deviation scores for age, cryptorchidism and micro-orchidism defined as testis volume <1 ml	(22)

### Clinical Evaluation

Auxologic data and past medical and family history were collected from medical records. Height and weight were expressed as standard deviation scores (SDS) using Argentine standards (25). Growth velocity was assessed considering a period of at least 6 months. Pubertal stage was assessed at the time of GHSTs according to Marshall and Tanner (26, 27). Penile size was compared to standardized data of the Argentine population (28).

### Hormone Measurements

Plasma GH was measured using a chemiluminescent immunometric assay (ICMA; Immulite 2000; Siemens Healthcare Diagnostics, Gwynedd, UK) at baseline, and 30, 45, 60 and 90 min after iv administration of arginine-HCl (0.5 g/kg), and 30, 60, 90, and 120 min following oral administration of clonidine (0.1 mg/m^2^) (29). Intra- and inter-assay coefficients of variation were <4%. GH standards were IS-80/505 from 2004 to 2011 (17) and rhGH IS 98/574 from 2012 to 2019 (18). Total IGF1 was measured by radioimmunoassay (30) and, since October 2009, by ICMA (Immulite 2000, Siemens) (31). Serum levels of T4, free T4, T3, TSH, cortisol, ACTH, and prolactin were determined by electrochemiluminescence (Elecsys Cobas e411; Roche, Indianapolis, IN, USA) (22, 32).

### Imaging

Pre- and post-gadolinium enhanced T1- and T2-weighted images of magnetic resonance imaging (MRI) studies of the brain and hypothalamic-pituitary region were evaluated. When MRI was not performed, it was considered as “missing value.”

### Prediction Model-Building Procedures and Statistical Analysis

We followed the TRIPOD guideline (16) for development, validation, and reporting of the proposed score. Most of the work was done using the KNIME software, version 3.7.0 (which is open source under GNU General Public License), and we also performed calculations using Microsoft^®^ Excel^®^ for Microsoft 365 MSO version 16.0.13001.20266 and GraphPad Prism^®^ version 8.4.3.

It is key to the analysis that we did not look for an unbiased model. The predictive criteria were intended to detect cases with GHD with the highest specificity. Type II errors (false negatives) were tolerated since, in real world practice, those cases would be subsequently diagnosed by the GHSTs. Conversely, type I errors (false positives) would have a very high impact since a patient would be diagnosed as having GHD without undergoing GHST and receive GH treatment. Therefore, the model should have the highest specificity (low rate of false positives) while keeping an acceptable sensitivity (rate of false negatives) to be clinically relevant. Since the predictors we considered were mostly binary, we could not construct ROC curves. Therefore, to develop and validate an accurate clinical prediction rule intended to diagnose GHD with the highest specificity in children without recurring to GHSTs, we used the following methodology (33, 34):

#### Step 1: Data Exploration

Data gathered from clinical experience and from existing criteria (1, 5) included the 15 dichotomous variables as potential predictors for diagnosing GHD without GHSTs defined in **Table 1**. We also included auxologic data (height and weight) and IGF1 and IGFBP3 serum levels, as continuous variables. Data exploration (cohort 2004–2014) consisted of: a) establishing linear dependencies between variables, using Pearson’s chi-squared test and product-moment coefficients for discrete and continuous variables respectively (a summary of all pairwise correlations is presented in **Supplementary Figure 1**); b) analyzing distributions of each continuous variable (distribution summary in **Supplementary Figure 2**), and c) establishing *a-priori* probabilities for GHD using a frequency table (**Supplementary Table 1**) for every variable (for instance, see *a-priori* for insipid diabetes in **Supplementary Table 2**, this analysis was done for each nominal variable). Data exploration and metrics used followed those previously described by Gelman (35). Finally, a model was automatically built using an information gain algorithm. We chose to construct a decision tree using the algorithm described by Quinlan (36). Decisions trees have the advantage that they have a graphical representation, and they give relevant information when inspected. The decision tree was subsequently used in the step 2 to select the predictors to be used in the final model.

#### Step 2: Feature Selection

Finally, we calculated conditional probabilities (from frequency tables) for the cases where two variables could give similar information. All variables selected by statistical means were checked from the point of view of clinical criteria.

Feature selection was done by combining statistical analyses with clinical criteria. First, from a quantitative point of view, we analyzed the decision tree built during step 1 and selected the variables that maximized entropy reduction. We also built a random forest to derive an analysis of feature relevance in order to validate the robustness of the set of conditions selected, as described (37). The algorithm used in KNIME to establish variable importance using random forests was the Tree Ensemble Learner (**Supplementary Table 3**), as previously established (38). Finally, all variables selected by statistical means were checked from the point of view of clinical criteria.

#### Step 3: Model Building

Model building was done in an iterative way: a model built using a machine learning algorithm was discussed from the point of view of clinical criteria. The model was then refined to build a new version. The process was iterated until the prediction rule was satisfactory from both points of view: the quantitative analysis and the clinical criteria.

We started by building a decision tree using the machine learning algorithm mentioned in step 2, fed only with the selected predictors. The result was discussed from the point of view of clinical criteria. The model was then refined to build a new version, by adjusting the parameters of the algorithm. The algorithm used in KNIME to build the decision trees was the Decision Tree Learner node. Some characteristics of this algorithm are as follows: numeric splits are always binary, dividing the domain in two partitions at a given split point. Nominal splits can be either binary or they can have as many outcomes as nominal values. The quality measure used for split calculation was the gain ration, no pos pruning method was used during the execution and the minimum number of records per node was set to 2. No root column was forced. We “pruned” the branch that had more false positives from the refined model, which led to lower sensitivity and higher specificity, which was the original goal. The final tree is shown in **Supplementary Figure 3**.

#### Step 4: Validation

In order to validate the prediction model, a dataset from a different cohort of patients (2017–2019) was used. This second cohort was independent from the first one, and the data set had not been used in the derivation of the model nor the analysis.

Validation included three axes: a) Safety, interpreted as no false positives. We aimed to keep type I error near 0 in the validation cohort; b) Usefulness, translated to sensitivity of the model, set at >0.2, meaning that the prediction rule would provide a diagnosis in at least 20% of all patients with suspected GHD, and c) significance: to establish significance, the null hypothesis H0 was that the proposed criterion did not imply GHD (as tested by GHSTs), and hence it was independent. We did not assume any further conditions nor particular distribution of the data. Statistical significance was not analyzed in the original dataset (2004–2014) since these data were used to derive the diagnostic procedure and to perform exploratory analysis of the features. For the validation data (2017–2019), we set an α-value of 0.00001 in order to build a very conservative model.

## Results

**Figure 1:**
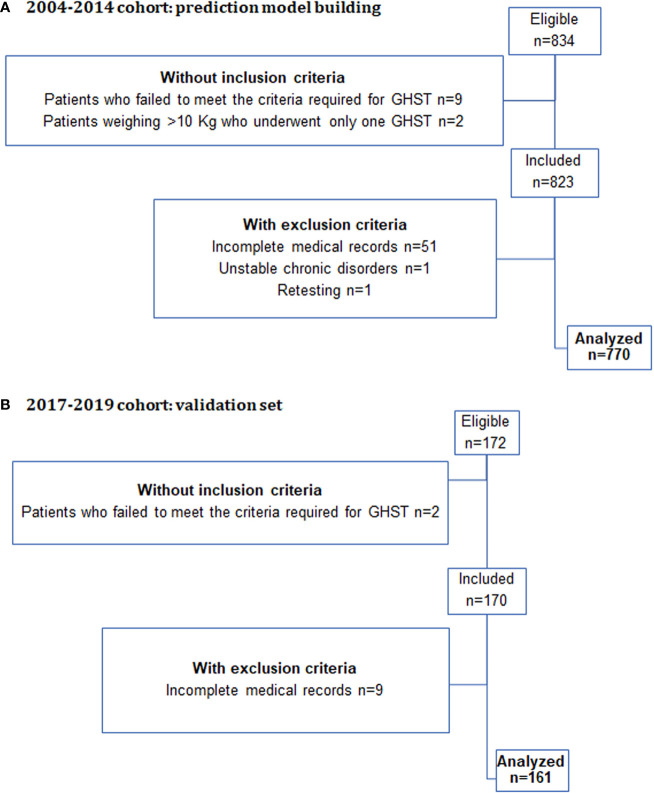
Flowchart of the selection process in the two cohorts of this study.

A total of 1,006 GHSTs were eligible for evaluation: 770 out of the 834 in the 2004–2014 cohort used to build the prediction model, and 161 of the 172 in the 2017–2019 cohort used to validate the model, could be analyzed (**Figure 1**).

Clinical characteristics of the 2004–2014 cohort are summarized in **Table 2**. It includes GHSTs performed in 491 boys and 279 girls (1.8:1), 79% were prepubertal, with a median age of 7.74 years and median height SDS of −2.51. Of the 770 GHSTs analyzed, 150 (19.5%) yielded results defined as GHD. The auxologic features were similar in patients without GHD (controls) and patients with GHD (cases), and within the latter, clinical features were similar in those predicted by the model and those not predicted. Both groups included congenital and acquired conditions (**Supplementary Table 4**). An MRI, used to define “pituitary dysgenesis” (**Table 1**) was available in 218 of the 770 patients (120 of 150 with GHD and 98 of 620 without GHD),

**Table 2:** Clinical characteristics of the 2004−2014 cohort, used for model building, classified according to growth hormone stimulation test (GHST) results.

Clinical characteristic		Total cohort	No GHD	GHD (all)	GHD (predicted by model)	GHD (not predictedby model)
No. (%)		770(100)	620(80.5)	150(19.5)	61(7.9 of total cohort;40.7 of all GHD)	89(11.6 of total cohort;59.3 of all GHD)
Gender M/F, No.(% M)		491/279(63.8)	403/217(65.0)	88/62(58.7)	41/20(67.2)	46/43(51.7)
Age at GHST median, years(IQ range)		7.74(5.22;11.08)	7.82(5.52; 11.11)	7.15(3.52; 10.89)	6.42(3.21; 11.85)	7.37(4.08; 10.16)
Pubertal stage at GHST, No.(%)	I	609 (79.1)	486 (78.4)	123 (82.0)	47 (77.0)	76 (85.4)
	II	99 (12.9)	83 (13.4)	16 (10.7)	8 (13.2)	8 (9.0)
	III	51 (6.6)	43 (6.9)	8 (5.3)	3 (4.9)	5 (5.6)
	IV	10 (1.3)	7 (1.1)	3 (2.0)	3 (4.9)	0 (0.0)
	V	1 (0.1)	1 (0.2)	0 (0.0)	0 (0.0)	0 (0.0)
Height SDS, median(IQ range)		−2.51(−3.02; −2.20)	−2.50(−2.98; −2.22)	−2.58(−3.18; −2.08)	−2.53(−3.39; −2.00)	−2.61(−3.06; −2.08)
Weight SDS, median(IQ range)		−2.30(−2.77; −1.71)	−2.34(−2.77; −1.81)	−2.03(−2.78; −0.74)	−1.87(−2.86;−0,14)	−2.07(−2.73; −1.15)
BMI SDS, median(IQ range)		−0.50(−0.94; −0.24)	−0.62(−0.96; −0.34)	0.00(−0.76; 0.51)	0.21(−0.53; 0.72)	−0.13(−0.87; 0.3)
IGHD/MPHD, No.(% IGHD)		−	−	86/64(57.3)	18/43(29.5)	82/7(92.1)
IGF1 SDS, median(IQ range)n		−1.86(−3.46; −0.59)657	−1.54(−3.11; −0.37)528	−2.92(−4.68, −1.77)129	−3.46(−4.68; −2.59)53	−2.59(−4.61; −1.23)76
IGFBP3 SDS, median(IQ range)n		−1.38(−2.28; −0.66)228	−1.21(−1.95; −0.62)190	−2.88(−3.32; −2.19)38	−3.22(−3.72; −2.50)19	−2.76(−3.09; −1.51)19

F, female; GHD, growth hormone deficiency; IGHD, isolated GHD; IQ, interquartile; M, male; MPHD, multiple pituitary hormone deficiency; SDS, standard deviation score.

In this cohort of 770 patients, after tuning the classification model trained using the 15 potential dichotomic predictors, we selected 9 variables of interest, with an odds ratio (OR) >5 and a p-value <0.0001 (**Table 3**); “cranial radiotherapy,” with an OR=4.4, was also selected given its clinical relevance. The continuous variables height, weight, and serum levels of IGF1 and IGFBP3 were not informative enough to be considered in the model (**Table 2**). We used a gain ratio, entropy reducing algorithm to automatically build a decision tree from the 10 selected variables, without reduce error pruning or limits on minimum number of records. Anterior pituitary hormone (TSH, ACTH, prolactin) deficiencies were categorized as 0 (no deficiency), 1 (any one deficiency), or ≥1 (multiple pituitary hormone deficiency) (**Supplementary Figure 3**). This model reached a 99.2% specificity (**Table 4**, “decision tree” column). Its sensitivity (49.3%) was above the required threshold, and overall accuracy was 89.5%, with a resulting F-measure = 0.646. Cross validation, with a test set of 20% of the cases randomly selected using stratified sampling, showed similar results, which remained consistent after repeating the random selection and changing the parameters (for instance, Gini index instead of gain ratio, or by using pruning). Alternative models, such as Naïve Bayes, also gave similar results.

**Table 3:** Categorical distribution and odds ratios for growth hormone deficiency of predictors used for model building (cohort 2004–2014).

Predictor	Stratum	Full cohort n=770	GHD n=150	No GHD n=620	OR (95% CI)	P value
Pituitary dysgenesis	Yes	38	38	0	∞ 13.2-∞	<0.0001
	No	180	82	98		
Clinical or radiological craniofacial midline abnormalities	Yes	36	19	17	5.1 2.6–10.4	<0.0001
	No	734	131	603		
Suprasellar or sellar tumor/surgery	Yes	16	13	3	19.5 5.6–64.8	<0.0001
	No	754	137	617		
Central nervous system infection	Yes	6	0	6	0.0 0.0–2.9	0.603
	No	764	150	614		
Severe traumatic brain injury	Yes	3	1	2	2.1 0.1–17.9	0.479
	No	767	149	618		
Cranial radiotherapy	Yes	20	10	10	4.4 1.8–10.6	0.002
	No	750	140	610		
Chemotherapy	Yes	21	10	11	4.0 1.7–9.0	0.003
	No	749	140	609		
Familial or sporadic GHD of genetic etiology	Yes	4	3	1	12.6 1.9–164.3	0.025
	No	766	147	619		
TSH deficiency	Yes	43	42	1	240.7 42.5–2,457.0	<0.0001
	No	727	108	619		
ACTH deficiency	Yes	32	32	0	∞ 43.3-∞	<0.0001
	No	738	118	620		
Prolactin deficiency	Yes	3	3	0	∞ 3.6-∞	<0.0001
	No	767	147	620		
Central diabetes insipidus	Yes	21	17	4	19.7 6.6–54.5	<0.0001
	No	749	133	616		
Neonatal hypoglycemia	Yes	30	18	12	6.9 3.4–14.5	<0.0001
	No	740	132	608		
Neonatal cholestatic jaundice	Yes	9	5	4	5.3 1.6–17.4	0.017
	No	761	145	616		
Neonatal hypogenitalism	Yes	26	18	8	10.4 4.4–23.0	<0.0001
	No	744	132	612		

CI, confidence interval; GHD, growth hormone deficiency; OR, odds ratio. P value of Fisher’s exact test.

**Table 4:** Performance of the prediction rule for diagnosing growth hormone deficiency (GHD).

	Decision tree (2004–2014 cohort)	Prediction rule (2004–2014 cohort)	Validation (2017–2019 cohort)
Specificity, % (95% CI)	99.2 (98.1–99.7)	100 (99.4–100)	99.2 (95.6–100)
Sensitivity, % (95% CI)	49.3 (41.5–57.3)	40.7 (33.1–48.7)	55.6 (39.6–70.5)
Positive PV, % (95% CI)	93.7 (86.0–97.3)	100 (94.1–100)	95.2 (77.3–99.8)
Positive LR	61.2	>1,000	69.4
NNT (95% CI)	1.21 (1.15–1.36)	1.14 (1.11–1.26)	1.19 (1.11–1.62)
Accuracy, %	89.5	88.4	89.4

LR, likelihood ratio; NNT, number needed to test with prediction rule to diagnose GHD; PV, predictive value.

The conditions “pituitary dysgenesis” and “≥1 anterior pituitary hormone (TSH, ACTH or prolactin) deficiency” were selected by the entropy reduction algorithms as the first variables to analyze. Interestingly, this selection was consistent with relevant clinical criteria. We also built a random forest (37) to derive an analysis of feature relevance, that reinforced the robustness of the set of conditions selected (**Supplementary Table 3**). Finally, we also calculated conditional probabilities for the cases where two variables could give similar information. The resulting prediction rule stated that a patient, who met the criteria required for performing GHSTs according to the *Summary Statement of the Growth Hormone Research Society* (5), was a case (GHD associated to risk factors) if (s)he met the following conditions:

1. Pituitary dysgenesis on MRI, or
2. Two or more anterior pituitary hormone (TSH, ACTH or prolactin) deficiencies, or
3. At least one anterior pituitary hormone (TSH, ACTH or prolactin) deficiency plus one of the following:
  - Neonatal symptoms of pituitary deficiency (hypoglycemia or hypogenitalism)
  - Central diabetes insipidus
  - Clinical or radiological craniofacial midline abnormalities
  - Suprasellar or sellar tumor/surgery
  - Cranial radiotherapy ≥18 Gy

The proposed criteria were very conservative for specificity (**Table 4**, “prediction rule” column), and missed 89 cases of GHD of the 2004−2014 cohort, which could then be diagnosed using GHSTs. Therefore, the proposed predictive rule diagnosed 61 GHD cases, which represents 40.7% of all GHD patients.

We validated the predictive rule in an independent cohort of patients (2017−2019). Clinical characteristics of this second cohort are summarized in **Table 5**. It includes GHSTs of 111 boys and 50 girls (2.2:1), 87% prepubertal, with a median age of 7.67 years and median height SDS of −2.57. This validation group was similar to the 2004−2014 cohort in terms of age, gender, or proportion of pathological tests compared, as well as in IGF1 serum levels. GHD was diagnosed in 36 patients (22.4%). An MRI was available in 46 of the 162 patients (27 of 36 with GHD and 19 of 126 without GHD), In this validation cohort (2017−2019), the prediction rule showed a specificity of 99.2%, and its positive predictive value was 95.2% (**Table 4**, “validation” column). The false-positive case according to our rule was a 10-year-old boy with height at −3.98 DS, IGF1 level 78 ng/ml (reference for age 60−370), who showed one peak GH level of 6.71 ng/ml at GHST, very close to the cutoff value, and no other remarkable feature. Given the severe growth deficiency, a therapeutic trial with GH treatment resulted in 1.06 DS gain in height after 1 year. GHD could not be ruled out in this patient despite the GHST result. The positive likelihood ratio of the prediction rule was 69.4, and the number needed to test with the rule was 1.19.

**Table 5:** Clinical characteristics of the 2017–2019 cohort, used to validate the predictive rule, classified according to Growth Hormone Stimulation Test (GHST) results.

Clinical characteristic		Total cohort	No GHD	GHD (all)	GHD (predicted by model)	GHD (Not predicted by model)
No. (%)		161 (100)	125 (77.6)	36 (22.4)	21 (13.0 of total cohort; 58.3 of all GHD)	15 (9.3 of total cohort; 41.7 of all GHD)
Gender M/F, No. (% M)		111/50 (68.9)	87/38 (69.6)	24/12 (66.7)	14/7 (66.7)	10/5 (66.7)
Age at GHST median, years (IQ range)		7.67 (5.1; 11.2)	7.64 (5.24; 11.02)	8.83 (3.73: 11.29)	6.32 (3.57; 11.16)	9.35 (5.49; 11.31)
Pubertal stage at GHST, n	I	140 (87.0)	109 (87.2)	31 (86.1)	20 (95.2)	10 (66.6)
	II	9 (5.6)	7 (5.6)	2 (5.6)	0 (0.0)	3 (20.0)
	III	11 (6.8)	9 (7.2)	2 (5.6)	1 (4.8)	1 (6.7)
	IV	1 (0.6)	0 (0.0)	1 (2.7)	0 (0.0)	1 (6.7)
	V	0 (0.0)	0 (0.0)	0 (0.0)	0 (0.0)	0 (0.0)
Height SDS, median (IQ range)		−2.57 (−3.07; −2.02);	−2.62 (−3.01; −2.18)	−2.35 (−3.69; −0.92)	−2.01 (−3.18; −0.37)	−2.54 (−3.70; −1.59)
Weight SDS, median (IQ range)		−2.20 (−2.80; −1.40)	−2.40 (−2.9; −1.7)	−1.25 (−2.13; 0.3)	−1.00 (−1.8; 0.6)	−2.00 (−2.8; 0.1)
BMI SDS, median (IQ range)		−0.30 (−0.88; −0.10)	−0.56 (−0.94; −0.28)	0.60 (−0.44; 1.10)	0.90 (−0.25; 1.20)	0.1 (−0.58; 0.45)
IGHD/MPHD, No. (%IGHD)		20/16 (55.6)	0/0	20/16 (55.6)	5/16 (23.8)	15/0 (100)
IGF1 SDS, median (IQ range)		−0.39 (−1.45; −0.43)	−0.09 (−1.02; 0.48)	−1.78 (−2.80; −0.50)	−2.07 (−2.78; −1.13)	−1.06 (−3.04; −0.32)

F, female; GHD, growth hormone deficiency; IGHD, Isolated GHD; IQ, interquartile; M, male; MPHD, multiple pituitary hormone deficiency; SDS, standard deviation score.

Finally, we tested significance following the methodology presented. The *a priori* probability of a GHD case in the second cohort was 36/161 (22.4%). We can safely assume that individual patients are independent cases, so we have a set of Bernoulli Trials under a binomial distribution. Thus, the p-value given by the cumulative function was <0.000001.

## Discussion

In this study, we identified clinically relevant risk factors for GHD in children, which were applied to build a robust clinical prediction rule to diagnose GHD, which could avoid resorting to GHSTs, in pediatric patients with growth failure and comorbidities. Our conclusion is based on the scientific evidence provided by the use of strict diagnostic criteria and clearly defined and accurately measured exposure variables in the analysis of a large cohort of GHSTs performed in a tertiary pediatric hospital.

The Endocrine Society clinical practice guideline on “Hypothalamic–Pituitary and Growth Disorders in Survivors of Childhood Cancer” advises using the same provocative testing to diagnose growth hormone deficiency in childhood cancer survivors as are used for diagnosing growth hormone deficiency in the non-cancer population as an ungraded good practice statement (11). On the other hand, the “Guidelines for the treatment of children with GHD, idiopathic short stature, and primary insulin-like growth factor 1 deficiency” of the Pediatric Endocrine Society (PES) suggest that in patients with auxological criteria, hypothalamic-pituitary defects and deficiency of at least one additional pituitary hormone, GHD diagnosis could potentially be established without performing GHSTs (1). However, due to the insufficient level of evidence according to the Grading of Recommendations, Assessment, Development, and Evaluation (GRADE) consensus (39), these recommendations could only be considered as conditional. Our study provides evidence to increase the strength of these recommendations.

We built a model on the knowledge generated by experts over the last 20 years (1, 5, 39)(and references therein), and applied a rigorous mathematical and machine-learning approach. Feature selection was based on the combination of statistical analyses with clinical criteria, used to refine the model in an iterative way until the prediction rule was satisfactory from both the statistical analysis and the clinical criteria, in a cohort of 770 GHSTs performed in our center between 2004 and 2014. Since the prediction rule was intended to diagnose GHD without the need for a GHST, and therefore type I errors would result in a false diagnosis of GHD leading to GH treatment in real world practice, we set goals of high specificity and positive predictive value for our rule. Of all the potential risk factors considered during model building, we identified the presence of pituitary dysgenesis on MRI or the existence of two or more anterior pituitary hormone deficiencies (TSH, ACTH, or prolactin) as specific enough to diagnose GHD without resorting to GHSTs in children meeting the criteria required for GHST by the Summary Statement of the Growth Hormone Research Society (5). Alternatively, if only one (TSH, ACTH, or prolactin) deficiency was present, the coexistence of central diabetes insipidus, neonatal symptoms of pituitary deficiency (hypoglycemia or hypogenitalism), sellar or suprasellar surgery or tumor (excluding microadenomas), clinical or radiological craniofacial midline abnormalities, or cranial radiotherapy ≥18 Gy, also led to a safe diagnosis. Gonadotropin deficiency was not considered in the analysis because its ascertainment may prove challenging in prepubertal patients.

To test the clinical applicability of the prediction rule, we validated our results using an independent cohort of 161 GHSTs performed between 2017 and 2019. Auspiciously, specificity was 99.2% in this second cohort, supporting the safety of our prediction rule. As expected, sensitivity was relatively low, reaching 55.6% in the validation cohort, indicating that almost half of the patients with GHD would only be identified after referring to GHSTs. Nonetheless, the sensitivity of the prediction rule applied to children meeting the criteria required for GHST by the Summary Statement of the Growth Hormone Research Society (5) would reduce in approximately half of the cases with GHD the need to perform a relatively invasive endocrine test, which underscores the clinical relevance of our results.

An unexpected, clinically relevant result is that only 20% of the children undergoing provocative tests, due to a suspicion of GHD, proved to be GH deficient (150 of 770 in the first cohort and 36 of 161 in the validation cohort). This is particularly significant given that only patients meeting the rigorous criteria defined by the Growth Hormone Research Society for prescribing GHSTs to children (5) were included in our study. This may be explained by the stringent criteria used to ascertain GHD in our center. Indeed, the diagnosis of GHD was based on peak GH levels <6.1 ng/ml between 2004 and 2011 (17), or <4.7 ng/ml between 2012 and 2019 (18), according to previously validated cutoff values (19).

Key strengths of this study are the high number of patients included in the construction of the predictive model as well as in the independent validation sample. It should also be stressed that strict criteria were used to define cases and controls: as mentioned above, the diagnosis of GHD was based on stringent cut-off levels for peak GH in GHSTs. A meticulous analysis of inclusion and exclusion criteria was performed to avoid inclusion bias. The population sample is representative of patients seeking advice from pediatric endocrinologists at referral centers for the assessment of short stature, which renders our results widely applicable.

Our study also has some limitations related to its design. Frequently, observational studies are prone to missing information in their datasets. To minimize memory bias, we limited the assessment of potential predictors to risk factors that were routinely reported in the clinical charts or were available from electronic records of endocrine laboratory or imaging study in our hospital. However, we cannot exclude that risk factors have been missed and, therefore, not included in the prediction rule we generated. Particularly, very few patients had a confirmed genetic diagnosis. This may explain why the condition “familial or sporadic GHD of genetic etiology” was not prioritized by our model. Also, MRI studies were not available in all patients; nonetheless, false positive prediction of GHD due to pituitary dysgenesis did not occur in any of the 98 controls (no GHD, **Table 3**) who underwent MRI.

In summary, this study developed an algorithm that led to the construction of a predictive rule for decision-making in the diagnosis of GHD in children typically seeking advice from pediatricians for growth failure, on the basis of a reduced number of clinically relevant and easily identifiable risk factors. The application of this rule avoids the need for GHSTs in a significant proportion of the patients in whom testing to assess GHD is presently indicated, and it is especially important for a subgroup of labile or vulnerable patients, such as infants, very low weight patients and children with oncological conditions or other comorbidities.

## Data Availability Statement

The original contributions presented in the study are included in the article/**Supplementary Material**. Further inquiries can be directed to the corresponding author.

## Ethics Statement

The studies involving human participants were reviewed and approved by Comité de Ética en Investigación, Hospital de Niños Ricardo Gutiérrez, Buenos Aires, Argentina. Written informed consent from the participants’ legal guardian/next of kin was not required to participate in this study in accordance with the national legislation and the institutional requirements.

## Author Contributions

FC, RG, DY, IB, GF, and RR conceived the study and designed the analysis plan. FC, DY, and RR did the statistical analyses. FC, RG, DY, GF, and RR wrote the manuscript. SMB, MS, MR, MB, AK, DB, PP, IB, and GF contributed to obtain and interpret the data. All authors contributed to the article and approved the submitted version.

## Funding

This work was partially supported by funding from: CONICET (Consejo Nacional de Investigaciones Científicas y Técnicas) grant PIP-11220130100687, and FONCYT (Fondo para la Investigación Científica y Tecnológica) grant PICT-2014-2490, Argentina.

## Conflict of Interest

DY is a founding partner of Practia S.A. GF is employed by Takeda Pharmaceutical. RR and IB report lecture honoraria from Novo Nordisk and non-financial support from Biosidus, Merck, Novo Nordisk, Pfizer, and Sandoz. RG reports lecture honoraria from Novo Nordisk and Raffo, and non-financial support from Merck, Novo Nordisk, Pfizer, and Sandoz.

The remaining authors declare that the research was conducted in the absence of any commercial or financial relationships that could be construed as a potential conflict of interest.
