# Supplementary material for: Development and Validation of a Prediction Rule for Growth Hormone Deficiency Without Need for Pharmacological Stimulation Tests in Children With Risk Factors
Source: Front Endocrinol (Lausanne). 2021 Feb 3;11:624684. doi: 10.3389/fendo.2020.624684 (PMC7887303; doi:10.3389/fendo.2020.624684)

**Supplementary Material**

**Supplementary Table 1.** A-priori probabilities for growth hormone deficiency (GHD) for each dichotomous variable used for model building. 0 = absence; 1 = presence; n = numer of patients.

| **Attribute** | **Value** | **GHD** | **n** | **A priori** |
| --- | --- | --- | --- | --- |
| (Supra)sellar tumour/surgery | 0 | 0 | 617 | 0.8013 |
| (Supra)sellar tumour/surgery | 0 | 1 | 134 | 0.1740 |
| (Supra)sellar tumour/surgery | 1 | 0 | 3 | 0.0039 |
| (Supra)sellar tumour/surgery | 1 | 1 | 16 | 0.0208 |
| ACTH deficiency | 0 | 0 | 620 | 0.8052 |
| ACTH deficiency | 0 | 1 | 118 | 0.1532 |
| ACTH deficiency | 1 | 0 | 0 | 0.0000 |
| ACTH deficiency | 1 | 1 | 32 | 0.0416 |
| CNS MRI | 0 | 0 | 522 | 0.6779 |
| CNS MRI | 0 | 1 | 30 | 0.0390 |
| CNS MRI | 1 | 0 | 98 | 0.1273 |
| CNS MRI | 1 | 1 | 120 | 0.1558 |
| CNS infection | 0 | 0 | 614 | 0.7974 |
| CNS infection | 0 | 1 | 150 | 0.1948 |
| CNS infection | 1 | 0 | 6 | 0.0078 |
| CNS infection | 1 | 1 | 0 | 0.0000 |
| Chemotherapy | 0 | 0 | 609 | 0.7909 |
| Chemotherapy | 0 | 1 | 140 | 0.1818 |
| Chemotherapy | 1 | 0 | 11 | 0.0143 |
| Chemotherapy | 1 | 1 | 10 | 0.0130 |
| Cranial radiotherapy | 0 | 0 | 610 | 0.7922 |
| Cranial radiotherapy | 0 | 1 | 140 | 0.1818 |
| Cranial radiotherapy | 1 | 0 | 10 | 0.0130 |
| Cranial radiotherapy | 1 | 1 | 10 | 0.0130 |
| Genetic GHD | 0 | 0 | 619 | 0.8039 |
| Genetic GHD | 0 | 1 | 147 | 0.1909 |
| Genetic GHD | 1 | 0 | 1 | 0.0013 |
| Genetic GHD | 1 | 1 | 3 | 0.0039 |
| Central Diabetes Insipidus | 0 | 0 | 616 | 0.8000 |
| Central Diabetes Insipidus | 0 | 1 | 133 | 0.1727 |
| Central Diabetes Insipidus | 1 | 0 | 4 | 0.0052 |
| Central Diabetes Insipidus | 1 | 1 | 17 | 0.0221 |
| Midline abnormalities | 0 | 0 | 603 | 0.7831 |
| Midline abnormalities | 0 | 1 | 131 | 0.1701 |
| Midline abnormalities | 1 | 0 | 17 | 0.0221 |
| Midline abnormalities | 1 | 1 | 19 | 0.0247 |
| Neonatal Cholestasis | 0 | 0 | 616 | 0.8000 |
| Neonatal Cholestasis | 0 | 1 | 145 | 0.1883 |
| Neonatal Cholestasis | 1 | 0 | 4 | 0.0052 |
| Neonatal Cholestasis | 1 | 1 | 5 | 0.0065 |
| Neonatal Hypogenitalism | 0 | 0 | 612 | 0.7948 |
| Neonatal Hypogenitalism | 0 | 1 | 132 | 0.1714 |
| Neonatal Hypogenitalism | 1 | 0 | 8 | 0.0104 |
| Neonatal Hypogenitalism | 1 | 1 | 18 | 0.0234 |
| Neonatal Hypoglycemia | 0 | 0 | 608 | 0.7896 |
| Neonatal Hypoglycemia | 0 | 1 | 132 | 0.1714 |
| Neonatal Hypoglycemia | 1 | 0 | 12 | 0.0156 |
| Neonatal Hypoglycemia | 1 | 1 | 18 | 0.0234 |
| Number of adenohypophysis deficiencies | 0 | 0 | 619 | 0.8039 |
| Number of adenohypophysis deficiencies | 0 | 1 | 102 | 0.1325 |
| Number of adenohypophysis deficiencies | 1 | 0 | 1 | 0.0013 |
| Number of adenohypophysis deficiencies | 1 | 1 | 21 | 0.0273 |
| Number of adenohypophysis deficiencies | 2 | 0 | 0 | 0.0000 |
| Number of adenohypophysis deficiencies | 2 | 1 | 25 | 0.0325 |
| Number of adenohypophysis deficiencies | 3 | 0 | 0 | 0.0000 |
| Number of adenohypophysis deficiencies | 3 | 1 | 2 | 0.0026 |
| One adenohypophysis deficiency | 0 | 0 | 619 | 0.8039 |
| One adenohypophysis deficiency | 0 | 1 | 102 | 0.1325 |
| One adenohypophysis deficiency | 1 | 0 | 1 | 0.0013 |
| One adenohypophysis deficiency | 1 | 1 | 48 | 0.0623 |
| Pituitary dysgenesis | 0 | 0 | 620 | 0.8052 |
| Pituitary dysgenesis | 0 | 1 | 112 | 0.1455 |
| Pituitary dysgenesis | 1 | 0 | 0 | 0.0000 |
| Pituitary dysgenesis | 1 | 1 | 38 | 0.0494 |
| Prolactin deficiency | 0 | 0 | 620 | 0.8052 |
| Prolactin deficiency | 0 | 1 | 147 | 0.1909 |
| Prolactin deficiency | 1 | 0 | 0 | 0.0000 |
| Prolactin deficiency | 1 | 1 | 3 | 0.0039 |
| TSH deficiency | 0 | 0 | 619 | 0.8039 |
| TSH deficiency | 0 | 1 | 108 | 0.1403 |
| TSH deficiency | 1 | 0 | 1 | 0.0013 |
| TSH deficiency | 1 | 1 | 42 | 0.0545 |
| Traumatic brain injury | 0 | 0 | 618 | 0.8026 |
| Traumatic brain injury | 0 | 1 | 149 | 0.1935 |
| Traumatic brain injury | 1 | 0 | 2 | 0.0026 |
| Traumatic brain injury | 1 | 1 | 1 | 0.0013 |

**Supplementary Table 2.** A-priori probabilities for central diabetes insipidus for each dichotomous variable used for model building, as an example. This analysis was repeated for each dichotomous variable replacing Diabtes insipidus with the relevant dichotomosu variable. 0 = absence; 1 = presence; n = numer of patients.

| **Attribute** | **Value** | **Diabetes insipidus** | **Count** | **A priori** |
| --- | --- | --- | --- | --- |
| (Supra)sellar tumour/surgery | 0 | 0 | 738 | 0.9584 |
| (Supra)sellar tumour/surgery | 0 | 1 | 13 | 0.0169 |
| (Supra)sellar tumour/surgery | 1 | 0 | 11 | 0.0143 |
| (Supra)sellar tumour/surgery | 1 | 1 | 8 | 0.0104 |
| ACTH deficiency | 0 | 0 | 728 | 0.9455 |
| ACTH deficiency | 0 | 1 | 10 | 0.0130 |
| ACTH deficiency | 1 | 0 | 21 | 0.0273 |
| ACTH deficiency | 1 | 1 | 11 | 0.0143 |
| CNS MRI | 0 | 0 | 552 | 0.7169 |
| CNS MRI | 0 | 1 | 0 | 0.0000 |
| CNS MRI | 1 | 0 | 197 | 0.2558 |
| CNS MRI | 1 | 1 | 21 | 0.0273 |
| CNS infection | 0 | 0 | 743 | 0.9649 |
| CNS infection | 0 | 1 | 21 | 0.0273 |
| CNS infection | 1 | 0 | 6 | 0.0078 |
| CNS infection | 1 | 1 | 0 | 0.0000 |
| Chemotherapy | 0 | 0 | 732 | 0.9506 |
| Chemotherapy | 0 | 1 | 17 | 0.0221 |
| Chemotherapy | 1 | 0 | 17 | 0.0221 |
| Chemotherapy | 1 | 1 | 4 | 0.0052 |
| Cranial radiotherapy | 0 | 0 | 731 | 0.9494 |
| Cranial radiotherapy | 0 | 1 | 19 | 0.0247 |
| Cranial radiotherapy | 1 | 0 | 18 | 0.0234 |
| Cranial radiotherapy | 1 | 1 | 2 | 0.0026 |
| GHD according to GHST | 0 | 0 | 616 | 0.8000 |
| GHD according to GHST | 0 | 1 | 4 | 0.0052 |
| GHD according to GHST | 1 | 0 | 133 | 0.1727 |
| GHD according to GHST | 1 | 1 | 17 | 0.0221 |
| Genetic GHD | 0 | 0 | 745 | 0.9675 |
| Genetic GHD | 0 | 1 | 21 | 0.0273 |
| Genetic GHD | 1 | 0 | 4 | 0.0052 |
| Genetic GHD | 1 | 1 | 0 | 0.0000 |
| Midline abnormalities | 0 | 0 | 717 | 0.9312 |
| Midline abnormalities | 0 | 1 | 17 | 0.0221 |
| Midline abnormalities | 1 | 0 | 32 | 0.0416 |
| Midline abnormalities | 1 | 1 | 4 | 0.0052 |
| Neonatal cholestasis | 0 | 0 | 740 | 0.9610 |
| Neonatal cholestasis | 0 | 1 | 21 | 0.0273 |
| Neonatal cholestasis | 1 | 0 | 9 | 0.0117 |
| Neonatal cholestasis | 1 | 1 | 0 | 0.0000 |
| Neonatal hypogenitalism | 0 | 0 | 725 | 0.9416 |
| Neonatal hypogenitalism | 0 | 1 | 19 | 0.0247 |
| Neonatal hypogenitalism | 1 | 0 | 24 | 0.0312 |
| Neonatal hypogenitalism | 1 | 1 | 2 | 0.0026 |
| Neonatal hypoglycemia | 0 | 0 | 721 | 0.9364 |
| Neonatal hypoglycemia | 0 | 1 | 19 | 0.0247 |
| Neonatal hypoglycemia | 1 | 0 | 28 | 0.0364 |
| Neonatal hypoglycemia | 1 | 1 | 2 | 0.0026 |
| Number of adenohypophysis deficiencies | 0 | 0 | 712 | 0.9247 |
| Number of adenohypophysis deficiencies | 0 | 1 | 9 | 0.0117 |
| Number of adenohypophysis deficiencies | 1 | 0 | 20 | 0.0260 |
| Number of adenohypophysis deficiencies | 1 | 1 | 2 | 0.0026 |
| Number of adenohypophysis deficiencies | 2 | 0 | 16 | 0.0208 |
| Number of adenohypophysis deficiencies | 2 | 1 | 9 | 0.0117 |
| Number of adenohypophysis deficiencies | 3 | 0 | 1 | 0.0013 |
| Number of adenohypophysis deficiencies | 3 | 1 | 1 | 0.0013 |
| One adenohypophysis deficiency | 0 | 0 | 712 | 0.9247 |
| One adenohypophysis deficiency | 0 | 1 | 9 | 0.0117 |
| One adenohypophysis deficiency | 1 | 0 | 37 | 0.0481 |
| One adenohypophysis deficiency | 1 | 1 | 12 | 0.0156 |
| Pituitary dysgenesis | 0 | 0 | 715 | 0.9286 |
| Pituitary dysgenesis | 0 | 1 | 17 | 0.0221 |
| Pituitary dysgenesis | 1 | 0 | 34 | 0.0442 |
| Pituitary dysgenesis | 1 | 1 | 4 | 0.0052 |
| Prolactin deficiency | 0 | 0 | 747 | 0.9701 |
| Prolactin deficiency | 0 | 1 | 20 | 0.0260 |
| Prolactin deficiency | 1 | 0 | 2 | 0.0026 |
| Prolactin deficiency | 1 | 1 | 1 | 0.0013 |
| TSH deficiency | 0 | 0 | 717 | 0.9312 |
| TSH deficiency | 0 | 1 | 10 | 0.0130 |
| TSH deficiency | 1 | 0 | 32 | 0.0416 |
| TSH deficiency | 1 | 1 | 11 | 0.0143 |
| Traumatic brain injury | 0 | 0 | 746 | 0.9688 |
| Traumatic brain injury | 0 | 1 | 21 | 0.0273 |
| Traumatic brain injury | 1 | 0 | 3 | 0.0039 |
| Traumatic brain injury | 1 | 1 | 0 | 0.0000 |

**Supplementary Table 3. Variable importance showing the relative importance of each attribute calculated from the forests by the variable-importance algorithm.**

| **Variable Name** | **Importance Level 0** | **Importance Level 1** |
| --- | --- | --- |
| Pituitary dysgenesis | 0·905 | 0·511 |
| Number of anterior adenohypophysis deficiencies | 0·905 | 0·684 |
| (Supra)sellar tumour/surgery | 0·517 | 0·422 |
| Central diabetes insipidus | 0·500 | 0·340 |
| Neonatal hypogenitalism | 0·455 | 0·175 |
| Familial or sporadic GHD of genetic aetiology | 0·308 | 0·200 |
| Neonatal Hypoglycaemia | 0·273 | 0·111 |
| Neonatal cholestatic jaundice | 0·143 | 0·167 |
| Traumatic brain injury | 0·111 | 0·078 |
| Cranial radiotherapy ≥18 Gy | 0·111 | 0·209 |
| Craniofacial midline abnormalities | 0·050 | 0·186 |
| CNS infection | 0·000 | 0·067 |
| Chemotherapy | 0·000 | 0·102 |

**Supplementary Table 4. Conditions associated with growth hormone deficiency (GHD) in the 2004-2014 and the 2017-2019 cohorts.***

|  | **Cohort 2004-2014**  (n = 150) | | | **Cohort 2017-2019**  (n = 36) | | |
| --- | --- | --- | --- | --- | --- | --- |
|  | **Total** | **IGHD** | **MPHD** | **Total** | **IGHD** | **MPHD** |
| **GHD predicted by the model** | **61** | **18** | **43** | **21** | **5** | **16** |
| **Congenital** |  | | | | | |
| Pituitary dysgenesis | 38 | 18 | 20 | 11 | 5 | 6 |
| Craniofacial midline abnormalities | 12 | 3 | 9 | 2 | 1 | 1 |
| **Acquired** |  | | | | | |
| Suprasellar or sellar tumour/surgery | 11 | 0 | 11 | 8 | 0 | 8 |
| Radiotherapy | 5 | 0 | 5 | 5 | 0 | 5 |
| Chemotherapy | 4 | 0 | 4 | 6 | 0 | 6 |
| Hypophysitis | 1 | 0 | 1 | 0 | 0 | 0 |
| Hydrocephalus | 1 | 0 | 1 | 0 | 0 | 0 |
| Histiocytosis | 2 | 0 | 2 | 0 | 0 | 0 |
| **Idiopathic** | 3 | 0 | 3 | 0 | 0 | 0 |
| **GHD not predicted by the model** | **89** | **82** | **7** | **15** | **15** | **0** |
| **Congenital** |  | | | | | |
| Anterior pituitary hypoplasia | 8 | 6 | 2 | 1 | 1 | 0 |
| Craniofacial midline abnormalities | 7 | 7 | 0 | 2 | 2 | 0 |
| Familial GHD | 3 | 2 | 1 | 0 | 0 | 0 |
| Prader Willi syndrome | 9 | 6 | 3 | 2 | 2 | 0 |
| Other syndromes ** | 5 | 5 | 0 | 2 | 2 | 0 |
| **Acquired** |  | | | | | |
| Suprasellar or sellar tumour/surgery | 5 | 5 | 0 | 1 | 1 | 0 |
| Radiotherapy | 5 | 5 | 0 | 3 | 3 | 0 |
| Chemotherapy | 6 | 6 | 0 | 2 | 2 | 0 |
| Traumatic brain injury | 1 | 1 | 0 | 0 | 0 | 0 |
| Hemosiderosis | 1 | 1 | 0 | 0 | 0 | 0 |
| **Idiopathic** | 47 | 46 | 1 | 6 | 6 | 0 |

IGHD: Isolated growth hormone deficiency, MPHD: Multiple pituitary hormone deficiency.

* Some patients have been included under two or more conditions (e.g. “Anterior pituitary hypoplasia” and “Craniofacial midline abnormalities” or “Suprasellar or sellar tumour/surgery”, “Radiotherapy” and “Chemotherapy”).

** No known association with GHD: Barter syndrome, Caudal regression syndrome, IMAGe syndrome, Klinefelter syndrome, Moebius syndrome, Turner syndrome, and X;14 translocation.

**Supplementary Figure 1. Pearson’s chi-squared test and product-moment coefficients for discrete and continuous variables respectively: predictors used in the model building cohort**. Color intensitiy indicates de correlation (corr.) values from -1 (red) to +1 (blue). n/a: not available.


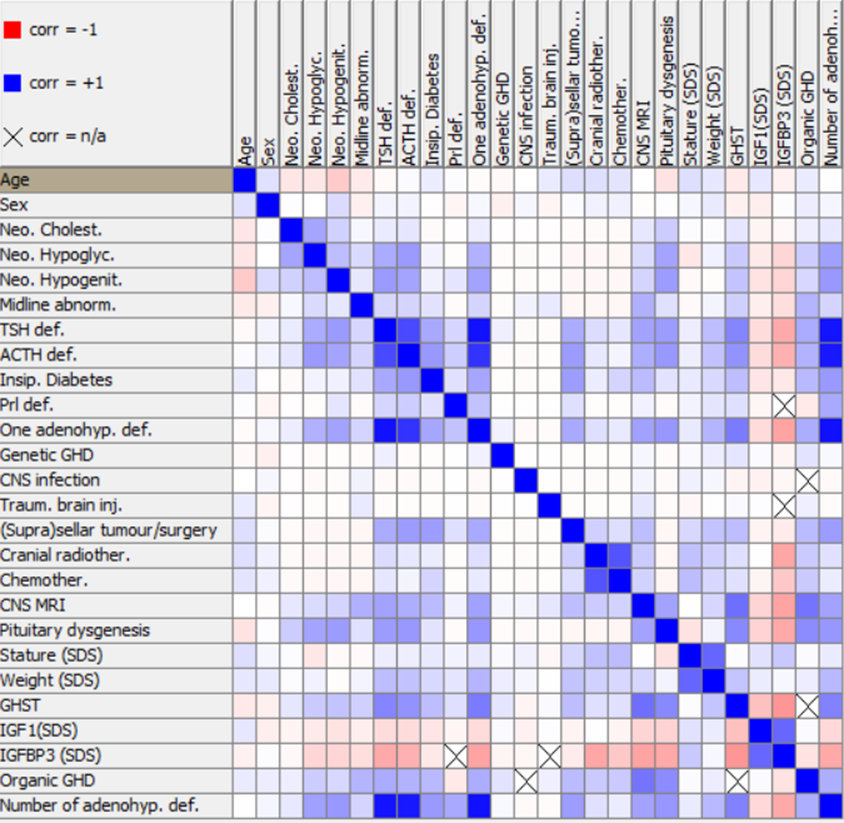


**Supplementary Figure 2. Distribution summary of continuous variables in the model building cohort**.

| Variable (SDS) | Mean | SD | Min | Max | Skewness | Kurtosis |  |
| --- | --- | --- | --- | --- | --- | --- | --- |
| Stature | -2.58 | 0.92 | -7.49 | 3.41 | 0.14 | 6.65 | 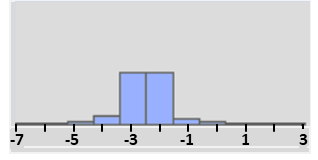 |
| Weight | -2.11 | 1.14 | -5.32 | 7.04 | 1.87 | 8.33 | 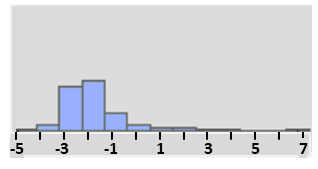 |
| IGF1 | -2.12 | 2.27 | -9.29 | 3.27 | -0.54 | 0.49 | 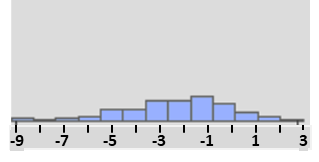 |
| IGFBP3 | -1.48 | 1.29 | -4.75 | 3.18 | 0.10 | 0.77 | 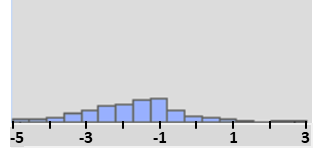 |

**Supplementary Figure 3. Random forest to derive the analysis of feature relevance: predictors used in the model building cohort.** % for “Total” refer to the total cohort (n=770).


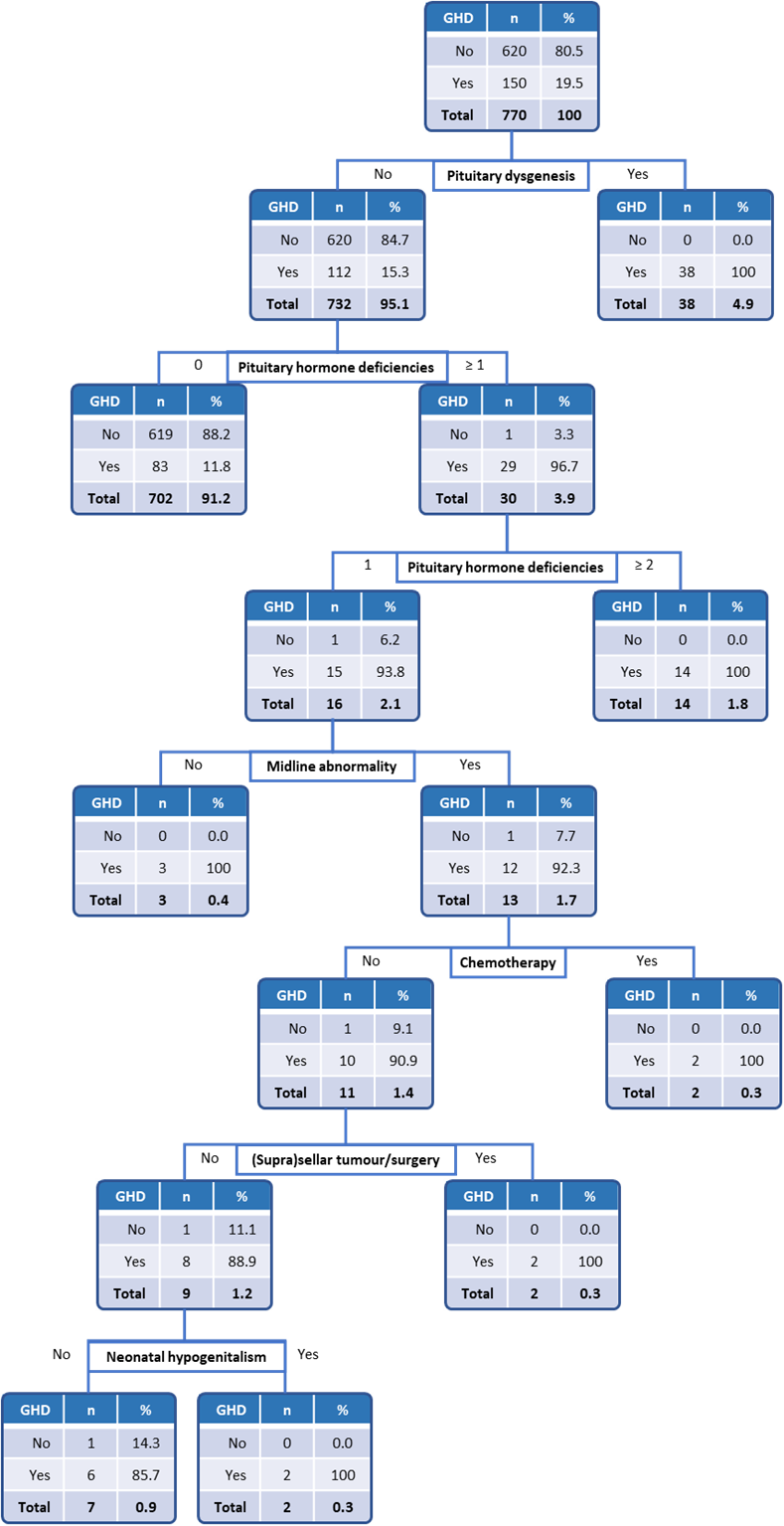

Supplement: Supplementary file 1 [file DataSheet_1.docx]
